# Optical Nerve Detection by Diffuse Reflectance Spectroscopy for Feedback Controlled Oral and Maxillofacial Laser Surgery

**DOI:** 10.1186/1479-5876-9-20

**Published:** 2011-02-10

**Authors:** Florian Stelzle, Azhar Zam, Werner Adler, Katja Tangermann-Gerk, Alexandre Douplik, Emeka Nkenke, Michael Schmidt

**Affiliations:** 1 Department of Oral and Maxillofacial Surgery, Erlangen University Hospital, Erlangen, Germany; 2 blz - Bavarian Laser Center, Erlangen, Germany; 3 Chair of Photonic Technologies, Friedrich-Alexander-University of Erlangen-Nuremberg, Erlangen, Germany; 4 SAOT - Graduate School in Advanced Optical Technologies, Friedrich-Alexander University of Erlangen-Nuremberg, Erlangen, Germany; 5 Department of Medical Informatics, Biometry and Epidemiology, Friedrich-Alexander University of Erlangen-Nuremberg, Erlangen, Germany

## Abstract

**Background:**

Laser surgery lacks haptic feedback, which is accompanied by the risk of iatrogenic nerve damage. It was the aim of this study to investigate diffuse reflectance spectroscopy for tissue differentiation as the base of a feedback control system to enhance nerve preservation in oral and maxillofacial laser surgery.

**Methods:**

Diffuse reflectance spectra of nerve tissue, salivary gland and bone (8640 spectra) of the mid-facial region of *ex vivo *domestic pigs were acquired in the wavelength range of 350-650 nm. Tissue differentiation was performed using principal component (PC) analysis followed by linear discriminant analysis (LDA). Specificity and sensitivity were calculated using receiver operating characteristic (ROC) analysis and the area under curve (AUC).

**Results:**

Five PCs were found to be adequate for tissue differentiation with diffuse reflectance spectra using LDA. Nerve tissue could be differed from bone as well as from salivary gland with AUC results of greater than 88%, sensitivity of greater than 83% and specificity in excess of 78%.

**Conclusions:**

Diffuse reflectance spectroscopy is an adequate technique for nerve identification in the vicinity of bone and salivary gland. The results set the basis for a feedback system to prevent iatrogenic nerve damage when performing oral and maxillofacial laser surgery.

## Introduction

Laser surgery provides several advantages. Lasers allow cutting biological tissue with high precision and minimal trauma. Furthermore, the ability to work remotely allows a high level of sterility [1-3]. However, these advantages come along with a lack of feedback: the surgeon does not receive sufficient information about the penetration depth of the laser cut or the type of tissue being ablated at the bottom of the laser cut. Hence, there is a risk of iatrogenic damage or the destruction of anatomical structures such as peripheral nerves [4-6].

Oral and maxillofacial surgery in particular has to deal with complex anatomy in the head and neck region, including major sensory and motor nerves. Damaging those can immensely affect function and aesthetics.

Two types of oral and maxillofacial surgeries are known for having an inherent risk of iatrogenic nerve damage, also in the case of conventional surgery techniques: First of all, removing the parotid gland can be accompanied by a damage of the facial nerve in 10 to 50% of cases. This leads to a temporary or permanent ipsilateral facial paralysis with an insufficient closure of eyelid and mouth [7,8]. Due to the fact that the branches of the facial nerve run directly through the parotid gland and both tissue types look very much alike, it is not easy for the surgeon to reliably differentiate the nerve from the gland. One opportunity for nerve identification is electrical stimulation. However, iatrogenic nerve damage could not even be significantly reduced by using advanced techniques of intra-operative neuromonitoring [9]. Secondly, orthognathic surgery performs a sagittal split through the lower jaw to correct the dental occlusion and the position of the mandible. The surgery can cause lesions to the lower alveolar nerve in 13 to 83% of the cases, with temporary or permanent numbness of the equilateral half of the lower lip and chin [10], which can hamper ingestion and speech production: The lower alveolar nerve runs through the mandible hidden in a bony canal. Consequently, cutting the bone has to be performed using specialized surgical techniques without a direct view on the nerve.

To benefit from laser cutting in oral and maxillofacial surgery and to simultaneously reduce iatrogenic nerve damage in the facial region, additional means are required that will automatically control the laser ablation through intra-operative nerve detection. Concerning the two surgeries mentioned above, there is to differentiate between nerves and salivary gland tissue as well as between nerves and bone. Several approaches for tissue specific laser ablation control using optical feedback systems have been described [11-13]. The basic idea is to regulate the laser ablation using optical tissue differentiation and to stop the laser cut when it reaches the vicinity of nerve tissue.

Diffuse Reflectance Spectroscopy (DRS) provides a relatively simple and cost-effective approach for tissue differentiation. The light applied is absorbed or scattered, depending on the optical properties of each tissue type. In the visible range, the main tissue absorbers are melanin and hemoglobin [14], and the main tissue scatterers are cell organelles (such as mitochondria, etc.) and cells [14]. Several types of normal healthy tissues from animals and humans have been described in terms of their optical properties by means of diffuse reflectance spectra *ex vivo *[15-17] and *in vivo *[18]. However, there is little information about the differentiation between different types of healthy tissue so far. Recently, the general feasability of optical tissue differentiation by a remote diffuse reflectance spectroscopy set up could be shown by our work group [19].

The goal of this study was to apply this diffuse reflectance spectroscopy technique on the differentiation between nerves and salivary gland tissue as well as between nerves and hard tissue. The differentiation between these tissue types is challenging due to their bioptic similarity. Also, it is highly relevant concerning the clinical application in the facial region, evolving the mentioned prior research and expanding it towards a clinical problem solution. The experiments deliver a basis for developing a remote optical nerve detection to control the surgical laser cut, with the intent of nerve preservation in oral and maxillofacial surgery.

## Materials and methods

### Tissue Samples

Four types of tissue - nerves, salivary glands, cancellous bone and cortical bone - were taken from 12 bisected *ex vivo *domestic pig heads (48 tissue samples). Nerve tissue samples were taken from the infraorbital nerve, salivary gland tissue was taken from the external part of the parotid gland and cancellous and cortical bone from the lower jaw in the region of the premolars. Hard tissue samples were cut with a water-cooled micro jigsaw, soft tissue samples were dissected using a scalpel. The bone and salivary gland tissue samples measured 5 × 5 cm with a thickness of 1 cm on average. The nerve tissue was provided with the perineural sheath and had a length of 5 cm and a diameter of 1 cm on average. After dissection, the tissue samples were carefully washed with a sterile saline solution to remove all superficial contamination, including clotted blood particles. We refrained from other cleaning methods, e.g. mechanical cleaning, to avoid alteration of the tissue samples. Until they were measured, the tissue samples were slightly moistened with a sterile saline solution and stored in a sealed box to avoid optical changes due to desiccation. All tissue samples were excised and measured on the day of slaughter within a maximum *ex vivo *time of 6 hours. Dissection, storage and measurements were conducted under a constant room temperature (22°C). There was no local or systemic illness of the animals to cause any pathological tissue alterations prior to sample extraction.

### Experimental Setup

**Figure 1:**
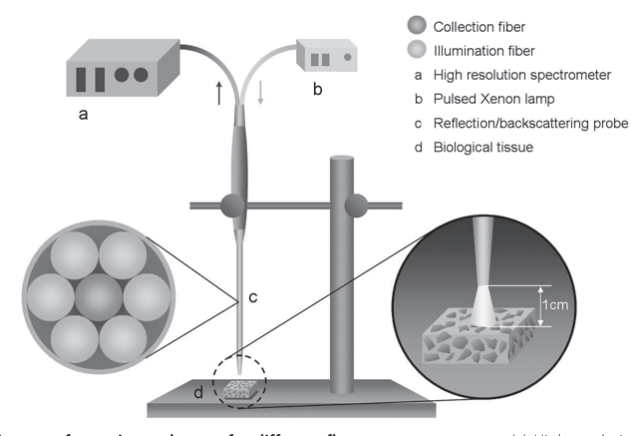
**Schematic diagram of experimental setup for diffuse reflectance measurement**. (a) High resolution spectrometer HR4000^® ^(1.1 nm optical resolution, Ocean Optics, USA), (b) Pulsed Xenon lamp PX-2^® ^(220-750 nm, Ocean Optics, USA), (c) Reflection/backscattering probe QR600-7-SR-125F^® ^(600 μm core, NA = 0.22, Ocean Optics, USA), (d) Biological tissue (pig *ex vivo*).

The diffuse reflectance of the tissues was measured *ex vivo *using a reflection/backscattering probe QR600-7-SR-125F^® ^(Ocean Optics, USA). The experimental setup shown in Figure 1 consisted of a Pulsed Xenon lamp PX-2^® ^(Ocean Optics, USA) projected onto tissue via the reflection/backscattering probe, and a high resolution spectrometer HR4000^® ^(Ocean Optics, USA) with a 1.1 nm optical resolution. The spectrometer has a dynamic range of 25 dB S/N and 31 dB. The spectrometer's accuracy is greater than 99%. The reflection/backscattering probe consists of 6 illumination fibers and a single collection fiber. Each optical fiber has a 600 μm core diameter and a 0.22 numerical aperture. The diffuse reflectance measurement was acquired within 10 ms of integration time. All tissue samples (12 tissue samples per tissue type) were placed and measured at a distance of 1 cm from the distal end of the probe. For each tissue sample, 6 different spots were chosen with a distance of 0.5 cm from each other. Per spot, 30 diffuse reflectance spectra were acquired (180 spectra per tissue sample). In total, 2160 diffuse reflectance spectra were acquired for each of the four types of tissue investigated.

The measurements were conducted under consistent conditions of minimized stray environmental light in the laboratory, which allowed us to exploit the experimental setup.

### Data Processing

The diffuse reflectance raw signal $S_{R_{d}}(\lambda )$ collected was converted into diffuse reflectance *R_d_*(λ). The light source emission spectrum reference spectrum was collected using the reflectance standard WS-1^® ^(250-1500 nm, Ocean Optics, USA). The diffuse reflectance was calculated as follows:

(1) $$R_{d}(\lambda )=S_{Rd}(\lambda )-S_{D}(\lambda )/S_{R}(\lambda )-S_{D}(\lambda )\cdot 100\%$$

Where:

*S_Rd _(λ)*: Diffuse reflectance raw signal (a.u.).

*S_R _(λ)*: Light source emission spectrum reference (a.u.).

*S_D _(λ)*: Background signal (a.u.).

Due to high noise in the near infrared spectral region, diffuse reflectance spectra beyond 650 nm were excluded from consideration. The background signal *S_D_*(λ) was used for the correction of stray light during measurement. After pre-processing, the spectra consisted of 1150 data points within the 350-650 nm range (0.26 nm wavelength resolution).

### Statistical analysis

We performed four consecutive steps for the statistical analysis of the data set. First, we reduced the number of variables using principal components analysis (PCA). A multiclass linear discriminant analysis (LDA) was trained with an appropriate number of principal components in the second step and class probabilities of observations not used for training were predicted in a third step. The last step included the calculation of the optimal threshold as well as sensitivity and specificity for tissue differentiation using receiver operating characteristic analysis (ROC). Our data set consisted of repeated measurements of only 48 specimens. Splitting the data into learning and test data was therefore inappropriate. Thus, we reused the data for training and testing by means of leave-one-out cross-validation, meaning that we split the data into 48 parts, each part consisting of all observations of a single specimen [20]. We then used 47 parts for calculating the PCA and training the LDA and the remaining part for testing. This was repeated for all 48 parts so that the predictions for all observations in the data set were estimated.

#### Principal Components Analysis (PCA)

To reduce the number of predictor variables, we performed a principal component analysis (PCA). In order to optimize the classification performance, we standardized and scaled the data: For each of the 1150 wavelength measurement values the mean of all measurements (at each specific wavelength) is subtracted from the measurement value and the result is divided by the standard deviation of all measurements (at each specific wavelength). Thus, a mean value of zero and a standard deviation of one were obtained for the measurements on a wavelength-by-wavelength basis. The PCA performs a decomposition of the data by creating orthogonal and thereby independent linear combinations of the variables, the so-called principal components (PC). There are as many PCs as variables, but the advantage is that only several are necessary to describe a large amount of the variation of the data, while the majority of the PCs' is responsible for less than 1% of the scatter. For our analysis, we used the first, second, fourth, fifth, and ninth principal component for classification. These principal components were determined: We performed a leave-one-out cross-validation to estimate the classification performance. In each cross-validation step, a PCA was calculated and Mann-Whitney U-tests were performed to test the discriminative power of each of the PCs between any pairwise tissue comparisons. For each of the pairwise comparisons, we selected those PCs that lead to the three lowest p-values, so that a maximum of 18 PCs was chosen if none of the PCs discriminated well between more than two tissues (6 pairwise comparisons) and a minimum of three PCs was chosen if the same three PCs discriminated best between all tissues. In the following steps we performed LDA training and testing and ROC analysis. We found that in each of the cross-validation steps, either nine or ten different PCs were selected, and that the first, second, fourth, fifth and ninth component was always selected while the remaining four or five selected components differed between individual cross-validation steps. Following our aim to build a classification method for practical use, we chose to repeat the cross-validation without the adaptive selection of the principal components, as this is prohibitive in a practical classification system. Instead, we trained and tested the LDA with only those PCs that were selected in each of the steps.

#### Classification

We utilized multiclass linear discriminant analysis (LDA) to separate the data (the five chosen principal components) with respect to their class membership, i.e., the tissue types [21]. Linear discriminant analysis is a method used to produce a discrimination rule that maximizes the ratio of interclass variance to intra-class variance of the observations. Instead of calculating fixed class memberships, we evaluated the class probabilities of each observation with respect to their inclusion as one of the four tissues.

#### Receiver Operating Characteristic (ROC) Analysis

The classification performance based on the estimated class probabilities was evaluated using receiver operating characteristic (ROC) analysis [22] with pairwise comparisons of all tissues: We calculated sensitivities and specificities for the optimal cutpoint maximizing the Youden index [23] which is defined as sensitivity + specificity - 1. This is the point with the largest distance to the diagonal and consequently is the optimal point of the ROC curve if sensitivity and specificity are of equal importance and without an existing restriction, e.g., a minimum specificity. Furthermore, we provided areas under the ROC curve (AUC).

The entire statistical analysis was carried out using the programming language R [24].

## Results

**Figure 2:**
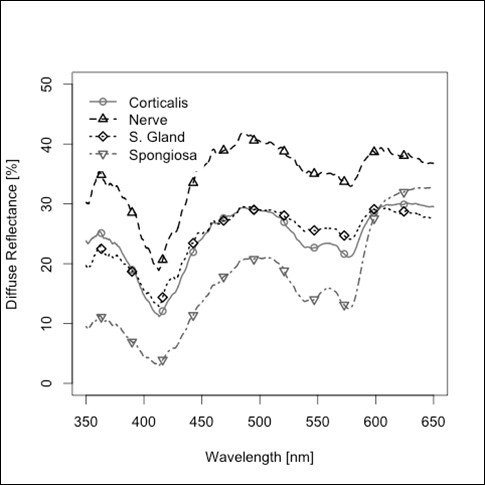
**Non-standardized diffuse reflectance spectra for different hard and soft tissues**.

**Figure 3:**
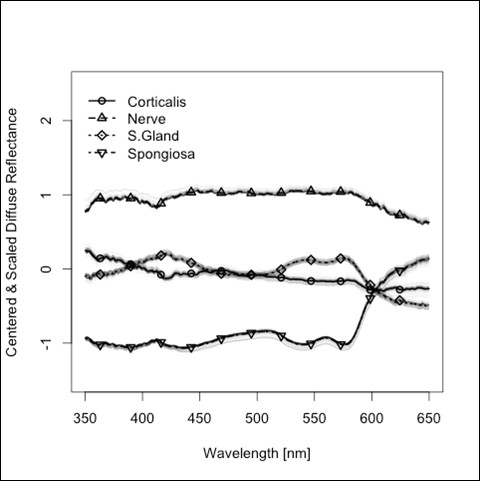
**Standardized diffuse reflectance spectra for different hard and soft tissues (Transformation of the entire data set using a mean of zero and a standard deviation of 1)**.

**Figure 4:**
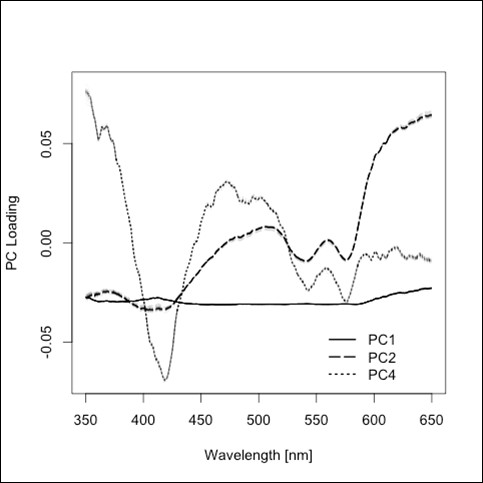
**PC loading**. PC 1 has a consistent contribution of loading along the investigated wavelength range; PC 2 and PC 4 show a higher variation of loading with prominent peaks at 410, 540 and 580 nm; PC 5 and 9 are more disturbed by the influence of noise (not shown in the figure).

The average spectra of diffuse reflectance in the wavelength range between 350 and 650 nm from the four types of tissue investigated in this study are shown in Figure 2 and in a normalized version in Figure 3. The 5 principal components (PCs) that were selected for classification were responsible for 97.34% of the variation of the data. The first PC does not show remarkable peaks and describes 88.06% of the variance of the diffuse reflectance spectra. PC 2 shows prominent peaks at 540 nm and 580 nm, PC 4 shows prominent peaks at 410 nm, 540 nm and 580 nm (Figure 4), but contributes only to 8.52% (PC 2) and 0.65% (PC 4) of the optical variance of the types of tissue. Starting with the fourth PC, chosen for the tissue differentiation (PC 5 and 9), the curves get more disturbed by the influence of noise.

**Figure 5:**
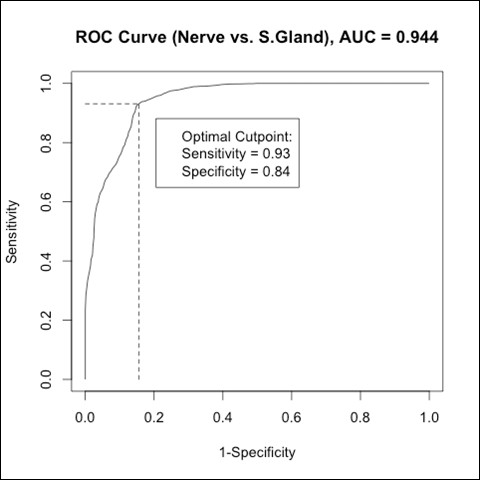
**ROC curve for comparison between nerve and salivary gland tissue (as example for the ROC-curves derived from the data analysis)**.

ROC analysis showed that PCA, followed by LDA, could differentiate between nerve tissue and salivary gland or osseous tissue types, respectively, with AUC results of 0.88 to 1.00 in this study (Table 1, Figure 5).

**Table 1:** AUC, Sensitivity and Specificity of tissue differentiation

	Cortical bone	Nerve	Salivary Gland
***AUC***			
**Nerve**	0.888		
**Salivary Gland**	0.894	0.944	
**Cancellous bone**	0.973	0.988	1.000
			
***Sensitivity***			
**Nerve**	0.837		
**Salivary Gland**	0.835	0.931	
**Cancellous bone**	0.913	0.919	1.000
			
***Specificity***			
**Nerve**	0.788		
**Salivary Gland**	0.808	0.844	
**Cancellous bone**	0.985	1.000	1.000

Concerning the sensitivity of differentiation between nerve tissue and hard tissue, the result was more than 90% for cancellous bone and 83% for cortical bone. The sensitivity of differentiation between nerve tissue and salivary gland tissue reached over 90% (Table 1). The specificity of tissue differentiation reached over 75% in all cases (Table 1).

## Discussion

For feedback controlled laser surgery, tissue differentiation is a crucial step. Especially in oral and maxillofacial surgery, the identification of major peripheral nerves is essential to avoid iatrogenic damage to these anatomical structures. Laser light may destroy neural structures through direct ablation or overheating due to laser cutting of adjacent tissue. Both can lead to reduced or missing nerve function [4-6].

In order to create the basis for optical nerve identification, we extracted diffuse reflectance spectra of 4 different types of tissue, i.e., nerve tissue, salivary gland tissue, cortical bone and cancellous bone from *ex vivo *pig samples. The selection of tissue types followed a clinical approach. Chosen were the tissue types which can be found on two typical oral and maxillofacial operations with a high risk of iatrogenic nerve damage: surgeries on the parotid gland jeopardize the facial nerve; orthognathic surgery on the lower jaw jeopardizes the lower alveolar nerve. To keep the experiments straightforward, only tissues in direct anatomical contact with the particular nerves were chosen for optical tissue differentiation. Five branches of the facial nerve go directly through the parotid gland and are fully surrounded by salivary gland tissue in the pre-auricular region [25]. The lower alveolar nerve runs through a mandibular canal and is directly surrounded by a bony canal. It is therefore surrounded by bone structure consisting of thin cortical bone. In rare cases of reduced or missing cortical bone the nerve may be directly surrounded by cancellous bone [26].

As the averaged spectra of the four tissues turned out not to be too distinct, advanced methods of analysis were used to differentiate the spectral curves. Analyzing the principal components of the spectra, we found 5 PCs that contributed significantly to the differentiation of the four types of tissue. These PCs were consistently chosen in all cross validation steps. PC 1 is responsible for more than 85% of the variance of the diffuse reflectance spectra derived from the four tissue types. For PC 1, the results of the PCA demonstrated a consistent contribution along the investigated wavelength range, without any remarkable peaks. PC 2 and PC 4 demonstrated prominent peaks at 410, 540 and 580 nm, which are meant to be related to the peaks of oxyhemoglobin and deoxyhemoglobin (Figure 4). Consequently, it is assumed that PC 1 provided information about the absorption/scattering contribution other than blood. This means that PC 1 can basically represent the bio-morphological variety of the tissues, such as size and number of cells and cell nuclei, cell organelles (e.g. mitochondria) and the amount and density of the extracellular matrix (ECM) including collagen. All of these are known to contribute to overall amounts of diffuse reflectance apart from blood [27-29]. The shape of the curve of PC 2 and PC 4 is similar to the spectral shape of blood, reflecting the contribution of blood absorption, reflection and backscattering in the visible range [30]. However, compared to PC 1, PC 2 and PC 4 are commonly responsible for only 9% of the variance between the reflectance spectra of different types of tissue.

Regarding the AUC results of the tissue differentiation, nerve tissue could be identified with a probability of 88.8% to 100%. The best result could be determined between salivary gland tissue and cancellous bone. However, it was not the major goal of this study to differentiate between these tissue types. The differentiation between nerves and cancellous bone reached high values of 98.8%. The lowest value was found for the differentiation between nerves and cortical bone (88.8%). An exclusively pairwise differentiation of tissue types may have yielded even better results. However, a pairwise differentiation of known tissue types does not meet the requirements of a clinical application with potential inter-individual variations of anatomy. Therefore, we chose a more complex mathematical approach implicating a multiclass analysis.

A high rate of correct tissue differentiation is only one part of nerve identification and preservation. The crucial step prior to a transfer of the technique to a clinical application may be the sensitivity of tissue differentiation. In this study, the sensitivity of nerve differentiation was found to be rather high with values ranging between 83.5% - 100%. The lowest result was achieved for the differentiation between cortical bone and salivary gland. However, the differentiation of that tissue pair was not a major goal of this study which focused on nerve tissue according to the clinical approach. A high sensitivity with over 90% could be demonstrated for the differentiation between nerves and cancellous bone as well as between nerves and salivary gland tissue. The differentiation of both tissue pairs is of high relevance considering clinical conditions. A similarly high result was achieved for the tissue pair nerves and cancellous bone yielding a specificity of tissue differentiation of 100%. The specificity of tissue differentiation between nerves and salivary gland tissue demonstrated only 84%, between nerves and cortical bone only 78%. However, a reduced specificity may be tolerated in favor of a high sensitivity if the aim is a precise and reliable preservation of major nerves. Additionally, a specificity of more than 70% may still allow for an uncomplicated performance in a clinical set-up.

Although it was not the goal of this study to investigate the differing optical properties of tissues, the differences of optical spectra may be explained by some considerations based on the similarity or the diversity of anatomical and biochemical structures: The single nerve fiber of peripheral nerves, like the infra-orbital nerve, is surrounded by a myelin sheath that contains 75% lipids (25% cholesterol, 20% galactocerebroside, 5% galactosulfatide, 50% phospholipids). Salivary gland tissue of humans and other mammals consists of epithelial cells, fibrous connective tissue and a high percentage of fat cells with 25% of volume on average [31]. The percentage of fat increases with age and can reach up to 60% of volume [32]. Lipocytes, the major cell population of fat tissue, predominantly consist of lipids. The compounds are triglycerides, cholesterol and other fatty acids [33]. It is assumed that the high proportion of lipids of both tissue types - salivary gland and the myelin sheath of peripheral nerves - leads to a similarity of optical properties, followed by a reduction of optical contrast of the derived diffuse reflectance spectra.

Bone tissue consists of 65% inorganic elements (calcium phosphate compounds, mainly hydroxyapatite [CA_10 _(PO_4_)_6 _(OH)_2_]) and 35% organic elements (collagen fibers, water, proteins). Cancellous bone demonstrates a porosity with an average of 80%, due to the intertrabecular marrow spaces [34]. Only 20% of the cancellous bone volume is built of bone tissue forming an osseous scaffold. The interspace of the osseous scaffold is filled with bone marrow. In adolescent beings, like the animals used in this study, the bone marrow is involved in the hematopoesis. Hence, it is highly vascular and mainly consists of hematopoietic cells and erythrocytes [35]. Therefore, the main optical properties of the cancellous bone are assumed to be constituted by hemoglobin and the hematopoietic cells. This may even be the case under *ex vivo *conditions, due to the fact that blood cells are fixed in the osseous scaffold of the cancellous bone. The fixation prevents the cells from descending to deeper tissue layers through gravity after circulatory arrest. After adolescence, aging is followed by a reduction of hematopoietic cells replaced by fat cells - indicating the transition from red to yellow bone marrow. Yellow bone marrow can contain an amount of fat cells of up to 80% [36]. That has to be kept in mind concerning a transition of the results to clinical conditions involving mature human beings.

Cortical bone demonstrates a very dense and homogeneous structure with a porosity of only 3.5% on average [34]. Therefore, the main optical properties of cortical bone are assumed to be constituted by the inorganic elements calcium and phosphate. In contrast, the reduced tissue differentiation between nerves and cortical bone does not reflect the biological diversity of the two tissue types. One possible explanation may be the reduced blood content of both tissues under *ex vivo *conditions, considering the fact that blood is known to be one major optical absorber and reflector of biological tissue [37]. Different types of tissue demonstrate different degrees of blood flow under *in vivo *conditions, which may considerably change the diffuse reflectance spectra [38]. In the presence of microcirculation, the blood content of the myelin sheath is different from the blood content of a salivary gland as well as of bone tissue. We expect that the discrimination algorithm based on diffuse reflectance spectra will work more reliably *in vivo *[29,39]. However, the findings have to be evaluated in further *in vivo *experiments.

For tissue differentiation a spectral range of 350-650 nm was used. Diffuse reflectance spectra over 650 nm showed high noise. Hence, the infrared spectral region was excluded in this study. The high noise may be a result of the presence of a large number of emission lines in the Xenon-lamp emission spectra or of the relatively low intensity of our light source. Other light sources, e.g., the tungsten halogen lamp, are known for a smooth emission profile showing favorable results for diffuse reflectance spectroscopy measurements [40,41]. On the other hand, using the Xenon light source yielded decent differentiation results providing sufficient intensity around the 410 nm peak - a wavelength which turned out to be of value for the differentiation of the tissue types investigated in this study. The results might be different considering the influence of blood. Hence, further research is necessary to investigate if the chosen set up is sufficient for the differentiation of biological tissue under *in vivo *conditions.

The results of this study were obtained using separated tissue samples containing only one type of tissue. One major challenge will be transferring the set up to a compound tissue sample which contains nerve tissue as well as other tissue types. Associated with this challenge is the penetration depth of the applied light, inheriting the possibility of optical spectra derived from several types of tissues situated together in the interrogation depth. Further research is necessary to establish a model simulation of the optical pathways to analyze nerve identification in biological tissue compounds.

In this study, we investigated tissue samples from domestic pigs. Extrapolating the results to human tissue, interspecies differences of optical tissue properties have to be considered. Additionally, there may be an alteration of optical tissue properties due to post mortem changes. It is known that de-oxygenation and a loss of hemoglobin are the main factors for a continuing decrease of absorption in the visible wavelength range during the first 24 hours post mortem [42]. Even if this process slows down after the first 24 hours, a further decrease of absorption may occur. To take the continuing post mortem changes of absorption decrease at the hemoglobin peaks into account, we kept the *ex vivo *time for tissue preparation and measurements as short as possible and, with 6 hours, equal for all the tissue types investigated in this study. However, post mortem changes may have altered the optical properties of the tissue samples, influencing the diffuse reflectance spectra.

A remote set-up was utilized for the measurements, to take two factors into account. Light delivery or measurement tools that are in direct contact with biological tissue may cause an alteration of optical properties due to a mechanical manipulation. The applied pressure on the tissue causes increased tissue absorption and scattering coefficients [43], which may alter the results of optical tissue differentiation. In addition, considering the clinical application of optical tissue differentiation, it has to be kept in mind that mechanical manipulation of the tissue may cause the spreading of germs or tumor cells during surgery [44,45]. Hence, focusing on a non-contact set-up, the environmental light has to be excluded as it may interfere with the optical spectra derived from the tissue. Executing the optical measurements in absolute darkness is a well known possibility [29,46], but does not meet the requirements for an uncomplicated clinical application. Hence, the diffuse reflectance spectra were mathematically corrected for environmental stray light to increase the signal to noise ratio during the measurements [47].

For a sufficient implementation of this tissue differentiation technique in a closed loop system to control laser ablation in surgery, the computational time required for analyzing the reflectance spectra is essential. A first basic feedback system was established with an acoustic sensor by our workgroup showing the general feasibility of a real-time sensor based control of laser surgery. The system was limited to the differentiation of two bone qualities [13,48]. Considering an expansion of the system towards a general applicability, it will be necessary to analyze several tissue types in a very short period of time which may be a mathematical and computational challenge. However, the time required for tissue differentiation was not the objective of our study. Before transferring the results of this study to a control system, this issue has to be investigated on further research.

## Conclusions

The results of this study show the general possibility of remote differentiation between nerve, salivary gland and bone tissue types, using diffuse reflectance spectroscopy. A control system can be established on the basis of this technology, which will be able to identify nerve tissue during oral and maxillofacial laser surgery to prevent iatrogenic nerve damage when performing surgeries on the parotid gland or the mandible. However, prior to any clinical application, further experiments are necessary to investigate the influence of blood microcirculation *in vivo*, the carbonization zone from laser ablation and/or bleeding on the surface of surgical wounds on diffuse reflectance tissue differentiation.

## Competing interests

The authors declare that they have no competing interests.

## Ethics considerations

Not necessary. The experimental study was carried out on tissues which were provided by a slaughterhouse.

## Authors' contributions

FS, AZ and KTG carried out the tissue preparation as well as the optical measurements. AZ, AD and KTG installed and adapted the optical set-up. WA participated in the design of the study and performed the statistical analysis. FS, AD, EN and MS performed the data analysis and assessment. FS and MS conceived of the study, participated in design and coordination and drafted the manuscript. All authors read and approved the final manuscript.
